# Desipramine hydro­chloride: a non-merohedrally twinned structure

**DOI:** 10.1107/S1600536810006203

**Published:** 2010-02-20

**Authors:** Jerry P. Jasinski, Ray J. Butcher, Q. N. M. Hakim Al-Arique, H. S. Yathirajan, A.R. Ramesha

**Affiliations:** a Department of Chemistry, Keene State College, 229 Main Street, Keene, NH 03435-2001, USA; b Department of Chemistry, Howard University, 525 College Street NW, Washington DC 20059, USA; c Department of Studies in Chemistry, University of Mysore, Manasagangotri, Mysore 570 006, India; d RL Fine Chem, Bangalore 560 064, India

## Abstract

The title compound, C_18_H_23_N_2_^+^·Cl^−^, is a non-merohedrally twinned salt [domains 0.9288 (3) and 0.0712 (3)] which crystallizes with four independent cation–anion pairs in the asymmetric unit. The seven-membered ring in each of the cations adopts a boat conformation, thus creating a butterfly effect within the ring system. The average value of the dihedral angle between the two aromatic rings in the four cations is 57.1 (1)°. The crystal packing is stabilized only slightly by a collection of inter­mediate N—H⋯Cl hydrogen-bonding inter­actions, which produce a weak, but cooperative, infinite, one-dimensional, inter­molecular hydrogen-bond network along the *a* axis. A MOPAC PM3 computational calculation gives support to these observations.

## Related literature

For related structures, see: Bindya *et al.* (2007 ▶); Butcher *et al.* (2007 ▶); Harrison *et al.* (2007 ▶); Klein *et al.* (1991 ▶, 1994 ▶); Portalone *et al.* (2007 ▶); Post *et al.* (1975 ▶); Swamy *et al.* (2007 ▶). For pharmaceutical uses of desipramine, see: Deupree *et al.* (2007 ▶); Cohen *et al.* (1990 ▶). For the analysis of desipramine hydro­chloride, see: Nagaraja *et al.* (2000 ▶) and for its use in the detection of trace amounts of blood in urine, see: Ahmed *et al.* (2002 ▶). For MOPAC PM3 calculations, see: Schmidt & Polik (2007 ▶).
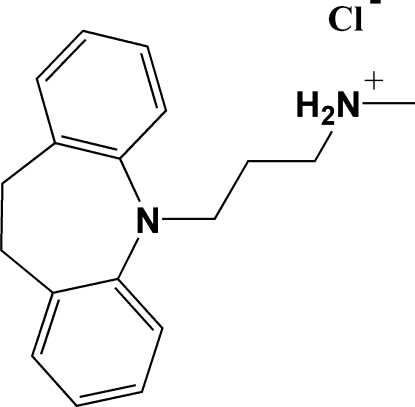


## Experimental

### 

#### Crystal data

C_18_H_23_N_2_^+^·Cl^−^
*M* *_r_* = 302.83
Triclinic, 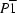

*a* = 10.7258 (3) Å
*b* = 15.9997 (6) Å
*c* = 20.6088 (8) Å
α = 107.347 (3)°
β = 89.960 (2)°
γ = 99.414 (2)°
*V* = 3326.0 (2) Å^3^
*Z* = 8
Mo *K*α radiation
μ = 0.23 mm^−1^
*T* = 110 K
0.53 × 0.44 × 0.32 mm

#### Data collection

Oxford Diffraction Xcalibur diffractometer with Ruby (Gemini Mo) detector
Absorption correction: multi-scan (*CrysAlis RED*; Oxford Diffraction, 2007 ▶) *T*_min_ = 0.806, *T*_max_ = 1.000
60082 measured reflections
60082 independent reflections
33887 reflections with *I* > 2σ(*I*)

#### Refinement

*R*[*F* ^2^ > 2σ(*F* ^2^)] = 0.083
*wR*(*F* ^2^) = 0.280
*S* = 1.09
60082 reflections
762 parameters
H-atom parameters constrained
Δρ_max_ = 1.40 e Å^−3^
Δρ_min_ = −0.93 e Å^−3^

### 

Data collection: *CrysAlis PRO* (Oxford Diffraction, 2007 ▶); cell refinement: *CrysAlis PRO*; data reduction: *CrysAlis PRO*; program(s) used to solve structure: *SHELXS97* (Sheldrick, 2008 ▶); program(s) used to refine structure: *SHELXL97* (Sheldrick, 2008 ▶); molecular graphics: *SHELXTL* (Sheldrick, 2008 ▶); software used to prepare material for publication: *SHELXTL*.

## Supplementary Material

Crystal structure: contains datablocks I. DOI: 10.1107/S1600536810006203/sj2729sup1.cif

Structure factors: contains datablocks I. DOI: 10.1107/S1600536810006203/sj2729Isup2.hkl

Additional supplementary materials:  crystallographic information; 3D view; checkCIF report

## Figures and Tables

**Table 1:** Hydrogen-bond geometry (Å, °)

*D*—H⋯*A*	*D*—H	H⋯*A*	*D*⋯*A*	*D*—H⋯*A*
N2*A*—H2*AB*⋯Cl3^i^	0.92	2.22	3.1186 (16)	165
N2*A*—H2*AC*⋯Cl3	0.92	2.17	3.0811 (17)	169
N2*B*—H2*BB*⋯Cl1	0.92	2.20	3.1027 (16)	168
N2*B*—H2*BC*⋯Cl4	0.92	2.20	3.1014 (16)	165
N2*C*—H2*CB*⋯Cl1	0.92	2.20	3.1069 (16)	167
N2*C*—H2*CC*⋯Cl4	0.92	2.21	3.1065 (16)	166
N2*D*—H2*DB*⋯Cl2	0.92	2.22	3.1176 (16)	166
N2*D*—H2*DC*⋯Cl2^ii^	0.92	2.18	3.0859 (16)	168

Symmetry codes: (i) 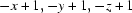; (ii) 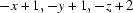.

**Fig. 1.**
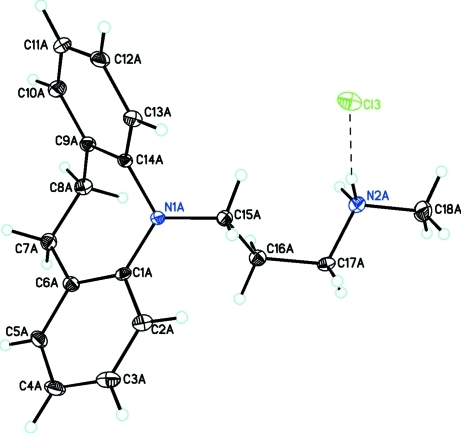
Molecular structure of cation-anion pair A for C18H23N2+.Cl-, (I), showing atom labeling scheme and 50% probability displacement ellipsoids. H atoms are presented as small circles of arbitrary radius.

**Fig. 2.**
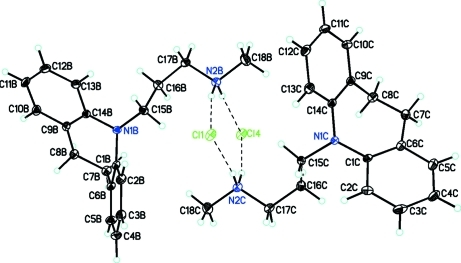
Molecular structure of C18H23N2+.Cl- showing one of two sets of a pair of enantiomers connected by a salt linkage between the proton that has been transfered from the hydrogen chloride to the desipramine (dp) group resulting in the protonation of the secondary-amine N atom of the side chain yielding an -NH2+- grouping and a chloride anion.

**Fig. 3.**
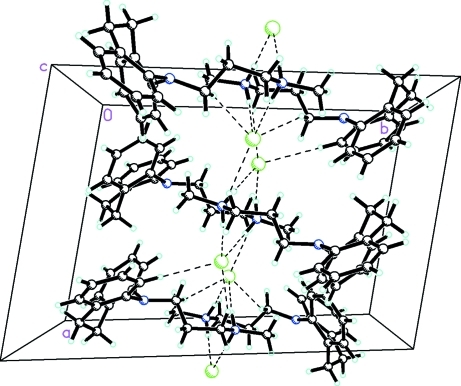
Packing diagram of the title compound, (I), viewed down the c axis. Dashed lines indicate weak intermediate intermolecular N—H···Cl interactions which produce an infinite network arranged along the c axis of the unit cell.

## supplementary crystallographic information

### Comment

The pharmacological properties of the active metabolites of the antidepressants desipramine and citalopram have been described (Deupree *et al.*, 2007). Desipramine, 10,11-dihydro-5-[3-(methylamino)propyl]-5*H*-dibenz[b,f]azepine is a tricyclic antidepressant (TCA) that inhibits the re-uptake of norepinephrine. Desipramine is an active metabolite of imipramine. Along with other tricyclics, desipramine has found use in treating neuropathic pain. The mechanism of action seems to involve the activation, through norepinephrine re-uptake inhibition, of descending pathways in the spinal cord that block pain signals from ascending to the brain. Desipramine is one of the most potent and selective medications in this respect. Desipramine hydrochloride works by increasing the amount of serotonin and norepinephrine in the brain so that the feeling of depression is prevented or relieved. By a separate mechanism, desipramine hydrochloride may also reduce pain related to peripheral neuropathy. The effect of desipramine hydrochloride on peripheral sympathetic nerve activity is reported (Cohen *et al.*, 1990). A sensitive spectrophotometric method has been developed for the determination of desipramine hydrochloride (Nagaraja *et al.*, 2000). Desipramine hydrochloride is proposed as a new reagent for detection of microamounts of blood in urine (Ahmed *et al.*, 2002).

The crystal structures of imipramine hydrochloride (Post *et al.* 1975), nortriptyline hydrochloride (Klein *et al.* 1991), amitriptyline hydrochloride (Klein *et al.* 1994), 5-[3-(dimethylamino)propyl]-10,11-dihydro-5*H*-dibenz[a,d][7]annulen-5-o l derived from the hydrolysis of amitriptyline (Portalone *et al.*, 2007) and amitriptylinium picrate (Bindya *et al.*, 2007) have been reported. The crystal structures of desipraminium picrate and desipraminium picrate hydrate have also been recently reported (Swamy *et al.*, 2007; Harrison *et al.*, 2007). In continuation of our work on related pharmaceutical compounds (Butcher *et al.*, 2007) and in view of the importance of desipramine, this paper reports the crystal structure of the title compound, C_18_H_23_N_2_^+^ . Cl^-^, (I).

The title compound, (I), is a non-merohedrally twinned salt and crystallizes with four independent cation-anion pairs (A,B,*C*,D) in the asymmetric unit cell. Cation-anion pair A is shown in Fig. 1. These cation-anion pairs form two sets of a pair of enantiomers connected by a salt linkage between the proton that has been transfered from the hydrogen chloride to the desipramine (dp) group resulting in the protonation of the secondary-amine N atom of the side chain yielding an -NH_2_^+^- grouping and a chloride anion (Fig. 2). The dihedral angle between the C1—C6 and C9—C14 ring planes in the cation is 57.5 (5)° (A), 57.8 (3)° (B), 55.8 (3)° (C) and 57.1 (9)° (D), respectively. The average value, 57.1 (1)°, is significantly higher than that observed in the related structures of desipraminum picrate, 52.79 (6)°, (Swamy *et al.*, 2007) and desipraminum picrate monohydrate, 53.92 (8)°, (Harrison *et al.*, 2007). The bond-angle sum for N1 is 353.44 (15)° (A), 353.08 (15)° (B), 353.98 (15)° (C) and 353.61 (15)° (D), respectively, which indicates a slightly distorted sp^2^ hybridization and a poorly aligned p orbital with the π cloud of adjacent benzene rings. The least squares plane of the C1/C14/C15/N1 group makes dihedral angles of 40.6 (8)° (A), 38.5 (4)° (B), 38.6 (1)° (C), 39.3 (8)° with the C1—C6 ring planes (average = 39.3 (1)°) and 57.7 (5)° (A), 56.1 (3)° (B), 57.1 (6)° (C), 56.8 (4)° (D) with the C9—C14 ring planes (average = 56.9 (7)°), respectively. In each of the four cations in the asymmetric unit the seven-membered ring approximates a boat thereby creating a butterfly effect within the ring system.

Crystal packing is stabilized only slightly by a collection of intermediate N—H···Cl hydrogen bonding interactions (Table 1) which produces a weak, but cooperative, infinite, one-dimensional, intermolecular hydrogen bond network along the *a* axis of the unit cell (Fig. 3).

From the results of a MOPAC PM3 computational calculation (Schmidt & Polik, 2007), the dihedral angle between the C1—C6 and C9—C14 ring planes in the cation is found to be 57.7 (3)°, just slightly higher than the 57.1 (1)° average value of the four molecules in the asymmetric unit. The least squares plane of the C1/C14/C15/N1 group makes dihedral angles of 40.09° with the C1—C6 ring plane and 55.60° with the C9—C14 ring plane, which is slightly higher than the C1—C6 ring plane average value in the crystal (39.3 (1)°) and slightly lower than the C9—C14 ring plane average value (56.9 (7)°) in the crystal. From these observations it is clear that the collective effect of the intermediate hydrogen bond interactions in (I) only slightly influences crystal packing stability, in sharp contrast to the traditional hydrogen bonding effects observed in desipraminium picrate (Swamy *et al.*, 2007) and desipraminium picrate monohydrate (Harrison *et al.*, 2007), respectively, which resulted in much greater deviations in these values.

### Experimental

The title compound was obtained as a gift sample from R.L. Fine Chem, Bangalore, India. The compound was used without further purification. X-ray quality crystals (m.p. 458-461 K) were obtained by slow evaporation from an aqueous solution.

### Refinement

The crystal is a non-merohedral twin with domains of 0.9288 (3) and 0.0712 (3). The structure was solved using the non-overlapping reflections and refined using all data in a SHELXTL HKLF5 format. In this format reflections cannot be merged hence the data appears to be more than 100% complete.

All of the H atoms were placed in their calculated positions and then refined using the riding model with N—H = 0.92, C—H = 0.95-0.99 Å, and with U_iso_(H) = 1.18-1.51U_eq_(C,*N*).

### Crystal data

**Table:**

C_18_H_23_N_2_^+^·Cl^−^	*Z* = 8
*M**_r_* = 302.83	*F*(000) = 1296
Triclinic, *P*1	*D*_x_ = 1.210 Mg m^−^^3^
Hall symbol: -P 1	Mo *K*α radiation, λ = 0.71073 Å
*a* = 10.7258 (3) Å	Cell parameters from 19411 reflections
*b* = 15.9997 (6) Å	θ = 4.8–32.7°
*c* = 20.6088 (8) Å	µ = 0.23 mm^−^^1^
α = 107.347 (3)°	*T* = 110 K
β = 89.960 (2)°	Chunk, colorless
γ = 99.414 (2)°	0.53 × 0.44 × 0.32 mm
*V* = 3326.0 (2) Å^3^	

### Data collection

**Table:**

Oxford Diffraction Xcalibur diffractometer with Ruby (Gemini Mo) detector	60082 independent reflections
Radiation source: Enhance (Mo) X-ray Source	33887 reflections with *I* > 2σ(*I*)
graphite	*R*_int_ = 0.0000
Detector resolution: 10.5081 pixels mm^-1^	θ_max_ = 32.9°, θ_min_ = 4.8°
ω scans	*h* = −15→16
Absorption correction: multi-scan (*CrysAlis RED*; Oxford Diffraction, 2007)	*k* = −23→24
*T*_min_ = 0.806, *T*_max_ = 1.000	*l* = −31→30
60082 measured reflections	

### Refinement

**Table:**

Refinement on *F*^2^	Primary atom site location: structure-invariant direct methods
Least-squares matrix: full	Secondary atom site location: difference Fourier map
*R*[*F*^2^ > 2σ(*F*^2^)] = 0.083	Hydrogen site location: inferred from neighbouring sites
*wR*(*F*^2^) = 0.280	H-atom parameters constrained
*S* = 1.09	*w* = 1/[σ^2^(*F*_o_^2^) + (0.1596*P*)^2^ + 0.0167*P*] where *P* = (*F*_o_^2^ + 2*F*_c_^2^)/3
60082 reflections	(Δ/σ)_max_ = 0.001
762 parameters	Δρ_max_ = 1.40 e Å^−^^3^
0 restraints	Δρ_min_ = −0.93 e Å^−^^3^

### Special details

**Table:**

Geometry. All esds (except the esd in the dihedral angle between two l.s. planes) are estimated using the full covariance matrix. The cell esds are taken into account individually in the estimation of esds in distances, angles and torsion angles; correlations between esds in cell parameters are only used when they are defined by crystal symmetry. An approximate (isotropic) treatment of cell esds is used for estimating esds involving l.s. planes.
Refinement. Refinement of *F*^2^ against ALL reflections. The weighted *R*-factor wR and goodness of fit *S* are based on *F*^2^, conventional *R*-factors *R* are based on *F*, with *F* set to zero for negative *F*^2^. The threshold expression of *F*^2^ > σ(*F*^2^) is used only for calculating *R*-factors(gt) etc. and is not relevant to the choice of reflections for refinement. *R*-factors based on *F*^2^ are statistically about twice as large as those based on *F*, and *R*- factors based on ALL data will be even larger.

### Fractional atomic coordinates and isotropic or equivalent isotropic displacement parameters (Å^2^)

**Table:**

	*x*	*y*	*z*	*U*_iso_*/*U*_eq_	
Cl1	0.21608 (4)	0.50056 (3)	0.74908 (3)	0.02317 (11)	
Cl2	0.28400 (4)	0.47582 (4)	0.99515 (3)	0.02684 (12)	
Cl3	0.70231 (4)	0.47109 (4)	0.49442 (3)	0.02741 (12)	
Cl4	−0.20743 (4)	0.50182 (4)	0.75046 (3)	0.02630 (11)	
N1A	0.62641 (14)	0.73248 (10)	0.67767 (8)	0.0178 (3)	
N2A	0.47318 (14)	0.44709 (10)	0.58136 (8)	0.0209 (3)	
H2AB	0.4135	0.4733	0.5663	0.025*	
H2AC	0.5472	0.4590	0.5607	0.025*	
N1B	0.12250 (14)	0.74135 (10)	0.68520 (8)	0.0184 (3)	
N2B	−0.00850 (14)	0.45333 (10)	0.64241 (8)	0.0200 (3)	
H2BB	0.0638	0.4729	0.6704	0.024*	
H2BC	−0.0754	0.4701	0.6682	0.024*	
N1C	0.00555 (14)	0.26122 (10)	0.81590 (8)	0.0198 (3)	
N2C	0.01723 (14)	0.54983 (10)	0.85746 (8)	0.0203 (3)	
H2CB	0.0796	0.5294	0.8294	0.024*	
H2CC	−0.0578	0.5338	0.8317	0.024*	
N1D	0.51015 (14)	0.26770 (10)	0.82101 (8)	0.0181 (3)	
N2D	0.50186 (14)	0.55342 (10)	0.91841 (8)	0.0199 (3)	
H2DB	0.4290	0.5289	0.9342	0.024*	
H2DC	0.5693	0.5393	0.9380	0.024*	
C1A	0.64897 (16)	0.77542 (12)	0.74864 (10)	0.0166 (4)	
C2A	0.71358 (18)	0.73653 (14)	0.78838 (11)	0.0246 (4)	
H2AA	0.7383	0.6807	0.7673	0.030*	
C3A	0.74191 (19)	0.77664 (16)	0.85651 (11)	0.0304 (5)	
H3AA	0.7876	0.7493	0.8815	0.036*	
C4A	0.70423 (19)	0.85697 (15)	0.88913 (11)	0.0299 (5)	
H4AA	0.7230	0.8852	0.9364	0.036*	
C5A	0.63804 (19)	0.89520 (14)	0.85063 (11)	0.0257 (4)	
H5AA	0.6117	0.9501	0.8728	0.031*	
C6A	0.60863 (16)	0.85672 (13)	0.78113 (10)	0.0196 (4)	
C7A	0.53339 (18)	0.90674 (13)	0.74724 (11)	0.0237 (4)	
H7AA	0.4574	0.9180	0.7736	0.028*	
H7AB	0.5857	0.9653	0.7515	0.028*	
C8A	0.48966 (17)	0.86384 (14)	0.67260 (11)	0.0236 (4)	
H8AA	0.4333	0.8064	0.6674	0.028*	
H8AB	0.4405	0.9029	0.6579	0.028*	
C9A	0.59992 (17)	0.84817 (13)	0.62775 (10)	0.0199 (4)	
C10A	0.6371 (2)	0.89616 (14)	0.58272 (11)	0.0277 (5)	
H10A	0.5923	0.9416	0.5798	0.033*	
C11A	0.7390 (2)	0.87870 (14)	0.54186 (11)	0.0294 (5)	
H11A	0.7622	0.9110	0.5106	0.035*	
C12A	0.80635 (19)	0.81377 (14)	0.54719 (11)	0.0262 (4)	
H12A	0.8770	0.8024	0.5201	0.031*	
C13A	0.77070 (17)	0.76544 (13)	0.59198 (10)	0.0213 (4)	
H13A	0.8164	0.7206	0.5951	0.026*	
C14A	0.66759 (16)	0.78258 (12)	0.63254 (9)	0.0163 (4)	
C15A	0.62178 (16)	0.63576 (12)	0.65202 (10)	0.0183 (4)	
H15A	0.6999	0.6204	0.6675	0.022*	
H15B	0.6169	0.6163	0.6016	0.022*	
C16A	0.50702 (17)	0.58786 (13)	0.67781 (11)	0.0219 (4)	
H16A	0.4297	0.6029	0.6610	0.026*	
H16B	0.5108	0.6106	0.7281	0.026*	
C17A	0.49516 (17)	0.48699 (13)	0.65663 (10)	0.0199 (4)	
H17A	0.5736	0.4711	0.6714	0.024*	
H17B	0.4239	0.4621	0.6796	0.024*	
C18A	0.4296 (2)	0.34976 (15)	0.56104 (13)	0.0407 (6)	
H18A	0.4896	0.3220	0.5799	0.061*	
H18B	0.4245	0.3259	0.5113	0.061*	
H18C	0.3459	0.3370	0.5785	0.061*	
C1B	0.16087 (16)	0.78628 (12)	0.75472 (10)	0.0167 (4)	
C2B	0.26179 (18)	0.76692 (13)	0.78704 (11)	0.0233 (4)	
H2BA	0.3086	0.7234	0.7620	0.028*	
C3B	0.29468 (19)	0.81052 (14)	0.85544 (11)	0.0267 (4)	
H3BA	0.3644	0.7975	0.8768	0.032*	
C4B	0.2259 (2)	0.87278 (14)	0.89219 (11)	0.0293 (5)	
H4BA	0.2464	0.9015	0.9393	0.035*	
C5B	0.1262 (2)	0.89333 (14)	0.86011 (11)	0.0273 (4)	
H5BA	0.0806	0.9374	0.8855	0.033*	
C6B	0.09191 (17)	0.85071 (13)	0.79162 (10)	0.0198 (4)	
C7B	−0.01698 (17)	0.86960 (14)	0.75529 (11)	0.0234 (4)	
H7BA	−0.0677	0.9058	0.7891	0.028*	
H7BB	−0.0723	0.8128	0.7310	0.028*	
C8B	0.02855 (18)	0.91882 (13)	0.70444 (11)	0.0231 (4)	
H8BA	−0.0467	0.9312	0.6835	0.028*	
H8BB	0.0793	0.9769	0.7301	0.028*	
C9B	0.10706 (16)	0.87295 (13)	0.64727 (10)	0.0188 (4)	
C10B	0.14031 (18)	0.91826 (14)	0.59927 (11)	0.0256 (4)	
H10B	0.1166	0.9748	0.6062	0.031*	
C11B	0.2061 (2)	0.88405 (15)	0.54239 (11)	0.0299 (5)	
H11B	0.2275	0.9166	0.5110	0.036*	
C12B	0.23969 (19)	0.80178 (15)	0.53218 (11)	0.0300 (5)	
H12B	0.2841	0.7770	0.4932	0.036*	
C13B	0.20918 (17)	0.75494 (13)	0.57840 (10)	0.0225 (4)	
H13B	0.2315	0.6977	0.5700	0.027*	
C14B	0.14582 (16)	0.79036 (12)	0.63743 (10)	0.0177 (4)	
C15B	0.12217 (17)	0.64509 (12)	0.66252 (10)	0.0200 (4)	
H15C	0.1205	0.6229	0.7026	0.024*	
H15D	0.2006	0.6329	0.6388	0.024*	
C16B	0.00773 (17)	0.59651 (13)	0.61475 (11)	0.0220 (4)	
H16C	−0.0701	0.6080	0.6393	0.026*	
H16D	0.0082	0.6211	0.5760	0.026*	
C17B	0.00322 (17)	0.49670 (13)	0.58728 (10)	0.0200 (4)	
H17C	−0.0697	0.4704	0.5540	0.024*	
H17D	0.0812	0.4848	0.5631	0.024*	
C18B	−0.0284 (2)	0.35501 (14)	0.61524 (13)	0.0350 (5)	
H18D	−0.1034	0.3340	0.5839	0.052*	
H18E	−0.0407	0.3294	0.6529	0.052*	
H18F	0.0459	0.3366	0.5909	0.052*	
C1C	0.00597 (16)	0.21325 (12)	0.86386 (9)	0.0166 (4)	
C2C	0.08932 (18)	0.24836 (14)	0.92181 (10)	0.0245 (4)	
H2CA	0.1421	0.3044	0.9287	0.029*	
C3C	0.0962 (2)	0.20309 (16)	0.96890 (11)	0.0319 (5)	
H3CA	0.1541	0.2279	1.0073	0.038*	
C4C	0.0192 (2)	0.12169 (15)	0.96061 (11)	0.0303 (5)	
H4CA	0.0250	0.0894	0.9921	0.036*	
C5C	−0.06634 (19)	0.08886 (14)	0.90512 (11)	0.0251 (4)	
H5CA	−0.1213	0.0341	0.9001	0.030*	
C6C	−0.07610 (17)	0.13206 (13)	0.85609 (10)	0.0192 (4)	
C7C	−0.17924 (17)	0.08723 (13)	0.79940 (11)	0.0222 (4)	
H7CA	−0.1579	0.0291	0.7731	0.027*	
H7CB	−0.2598	0.0750	0.8211	0.027*	
C8C	−0.20272 (17)	0.13679 (14)	0.74909 (10)	0.0228 (4)	
H8CA	−0.2734	0.1014	0.7165	0.027*	
H8CB	−0.2278	0.1942	0.7741	0.027*	
C9C	−0.08723 (17)	0.15374 (12)	0.71060 (10)	0.0182 (4)	
C10C	−0.0802 (2)	0.11172 (14)	0.64149 (10)	0.0261 (4)	
H10C	−0.1503	0.0698	0.6171	0.031*	
C11C	0.0282 (2)	0.13006 (14)	0.60735 (11)	0.0273 (4)	
H11C	0.0312	0.1015	0.5600	0.033*	
C12C	0.13166 (19)	0.19016 (14)	0.64277 (11)	0.0260 (4)	
H12C	0.2062	0.2021	0.6198	0.031*	
C13C	0.12624 (17)	0.23284 (13)	0.71167 (11)	0.0236 (4)	
H13C	0.1972	0.2740	0.7359	0.028*	
C14C	0.01636 (16)	0.21541 (12)	0.74571 (9)	0.0167 (4)	
C15C	0.05277 (17)	0.35747 (12)	0.83755 (10)	0.0199 (4)	
H15E	0.0622	0.3788	0.7971	0.024*	
H15F	0.1370	0.3700	0.8614	0.024*	
C16C	−0.03885 (17)	0.40641 (13)	0.88492 (11)	0.0227 (4)	
H16E	−0.1217	0.3953	0.8599	0.027*	
H16F	−0.0517	0.3818	0.9236	0.027*	
C17C	0.00607 (17)	0.50606 (13)	0.91257 (10)	0.0213 (4)	
H17E	−0.0542	0.5326	0.9456	0.026*	
H17F	0.0895	0.5176	0.9370	0.026*	
C18C	0.0473 (2)	0.64785 (14)	0.88395 (13)	0.0357 (5)	
H18G	−0.0151	0.6698	0.9166	0.054*	
H18H	0.0450	0.6731	0.8462	0.054*	
H18I	0.1320	0.6657	0.9066	0.054*	
C1D	0.51377 (16)	0.22531 (12)	0.74997 (9)	0.0160 (3)	
C2D	0.59751 (17)	0.26645 (14)	0.71156 (10)	0.0237 (4)	
H2DA	0.6476	0.3227	0.7334	0.028*	
C3D	0.60846 (19)	0.22704 (16)	0.64306 (11)	0.0305 (5)	
H3DA	0.6659	0.2560	0.6183	0.037*	
C4D	0.5352 (2)	0.14472 (16)	0.61007 (11)	0.0309 (5)	
H4DA	0.5438	0.1162	0.5631	0.037*	
C5D	0.44992 (18)	0.10531 (14)	0.64707 (10)	0.0239 (4)	
H5DA	0.3984	0.0499	0.6243	0.029*	
C6D	0.43638 (16)	0.14369 (12)	0.71671 (10)	0.0183 (4)	
C7D	0.33540 (18)	0.09215 (13)	0.74909 (11)	0.0235 (4)	
H7DA	0.3598	0.0337	0.7448	0.028*	
H7DB	0.2549	0.0806	0.7219	0.028*	
C8D	0.30945 (18)	0.13415 (14)	0.82366 (11)	0.0247 (4)	
H8DA	0.2797	0.1910	0.8287	0.030*	
H8DB	0.2413	0.0942	0.8375	0.030*	
C9D	0.42521 (18)	0.15095 (13)	0.86956 (10)	0.0221 (4)	
C10D	0.4375 (2)	0.10296 (14)	0.91468 (12)	0.0300 (5)	
H10D	0.3708	0.0568	0.9165	0.036*	
C11D	0.5457 (2)	0.12130 (14)	0.95734 (12)	0.0316 (5)	
H11D	0.5517	0.0889	0.9888	0.038*	
C12D	0.6448 (2)	0.18731 (14)	0.95360 (11)	0.0279 (5)	
H12D	0.7194	0.1995	0.9820	0.033*	
C13D	0.63488 (18)	0.23548 (13)	0.90830 (10)	0.0220 (4)	
H13D	0.7026	0.2806	0.9057	0.026*	
C14D	0.52467 (17)	0.21739 (12)	0.86643 (10)	0.0176 (4)	
C15D	0.55222 (16)	0.36414 (12)	0.84757 (10)	0.0188 (4)	
H15G	0.6383	0.3799	0.8326	0.023*	
H15H	0.5559	0.3827	0.8980	0.023*	
C16D	0.46105 (17)	0.41302 (13)	0.82192 (11)	0.0222 (4)	
H16G	0.4533	0.3904	0.7716	0.027*	
H16H	0.3764	0.3988	0.8390	0.027*	
C17D	0.50053 (17)	0.51341 (13)	0.84319 (10)	0.0198 (4)	
H17G	0.5860	0.5283	0.8273	0.024*	
H17H	0.4411	0.5392	0.8212	0.024*	
C18D	0.5113 (2)	0.65135 (15)	0.93995 (13)	0.0399 (6)	
H18J	0.5856	0.6777	0.9207	0.060*	
H18K	0.5196	0.6743	0.9897	0.060*	
H18L	0.4349	0.6668	0.9237	0.060*	

### Atomic displacement parameters (Å^2^)

**Table:**

	*U*^11^	*U*^22^	*U*^33^	*U*^12^	*U*^13^	*U*^23^
Cl1	0.01509 (19)	0.0342 (3)	0.0256 (3)	0.00686 (18)	0.00291 (16)	0.0158 (2)
Cl2	0.01409 (19)	0.0427 (3)	0.0228 (3)	0.00182 (19)	0.00046 (16)	0.0101 (2)
Cl3	0.0195 (2)	0.0429 (3)	0.0234 (3)	0.0157 (2)	0.00421 (17)	0.0099 (2)
Cl4	0.0160 (2)	0.0403 (3)	0.0278 (3)	0.00850 (19)	0.00336 (17)	0.0162 (2)
N1A	0.0245 (8)	0.0154 (7)	0.0146 (8)	0.0052 (6)	0.0025 (6)	0.0052 (7)
N2A	0.0180 (7)	0.0232 (8)	0.0226 (9)	0.0027 (6)	−0.0022 (6)	0.0091 (7)
N1B	0.0269 (8)	0.0156 (7)	0.0137 (8)	0.0065 (6)	0.0001 (6)	0.0046 (7)
N2B	0.0168 (7)	0.0228 (8)	0.0211 (9)	0.0034 (6)	−0.0001 (6)	0.0079 (7)
N1C	0.0263 (8)	0.0177 (8)	0.0137 (8)	−0.0017 (6)	0.0024 (6)	0.0051 (7)
N2C	0.0177 (7)	0.0248 (8)	0.0205 (9)	0.0064 (6)	0.0029 (6)	0.0084 (7)
N1D	0.0249 (8)	0.0165 (7)	0.0136 (8)	0.0029 (6)	−0.0006 (6)	0.0059 (7)
N2D	0.0178 (7)	0.0239 (8)	0.0208 (9)	0.0069 (6)	0.0042 (6)	0.0090 (7)
C1A	0.0156 (8)	0.0202 (9)	0.0156 (9)	0.0005 (7)	0.0033 (6)	0.0092 (8)
C2A	0.0250 (9)	0.0299 (11)	0.0224 (11)	0.0067 (8)	0.0034 (8)	0.0120 (9)
C3A	0.0292 (10)	0.0434 (13)	0.0227 (12)	0.0062 (9)	−0.0005 (8)	0.0162 (11)
C4A	0.0329 (11)	0.0382 (12)	0.0148 (11)	−0.0039 (9)	−0.0001 (8)	0.0076 (10)
C5A	0.0297 (10)	0.0243 (10)	0.0176 (11)	−0.0001 (8)	0.0033 (8)	0.0006 (9)
C6A	0.0175 (8)	0.0226 (9)	0.0176 (10)	0.0015 (7)	0.0029 (7)	0.0052 (8)
C7A	0.0242 (9)	0.0219 (10)	0.0248 (11)	0.0050 (8)	0.0043 (8)	0.0060 (9)
C8A	0.0208 (9)	0.0279 (10)	0.0257 (11)	0.0082 (8)	−0.0005 (7)	0.0115 (9)
C9A	0.0204 (9)	0.0213 (9)	0.0184 (10)	0.0027 (7)	−0.0003 (7)	0.0068 (8)
C10A	0.0376 (11)	0.0246 (10)	0.0252 (12)	0.0091 (9)	0.0022 (9)	0.0118 (10)
C11A	0.0432 (12)	0.0234 (10)	0.0224 (12)	0.0001 (9)	0.0057 (9)	0.0113 (9)
C12A	0.0267 (10)	0.0299 (11)	0.0180 (11)	−0.0003 (8)	0.0063 (8)	0.0040 (9)
C13A	0.0221 (9)	0.0246 (10)	0.0182 (10)	0.0071 (8)	0.0015 (7)	0.0062 (8)
C14A	0.0167 (8)	0.0182 (9)	0.0132 (9)	0.0000 (7)	−0.0018 (6)	0.0050 (8)
C15A	0.0183 (8)	0.0185 (9)	0.0192 (10)	0.0057 (7)	0.0053 (7)	0.0060 (8)
C16A	0.0199 (9)	0.0242 (10)	0.0224 (10)	0.0044 (7)	0.0049 (7)	0.0079 (9)
C17A	0.0187 (8)	0.0252 (10)	0.0188 (10)	0.0034 (7)	0.0036 (7)	0.0113 (8)
C18A	0.0579 (15)	0.0249 (11)	0.0332 (14)	−0.0072 (10)	−0.0118 (11)	0.0073 (11)
C1B	0.0173 (8)	0.0186 (9)	0.0171 (10)	0.0047 (7)	0.0028 (6)	0.0090 (8)
C2B	0.0231 (9)	0.0269 (10)	0.0231 (11)	0.0075 (8)	0.0027 (7)	0.0107 (9)
C3B	0.0268 (10)	0.0303 (11)	0.0258 (12)	0.0020 (8)	−0.0062 (8)	0.0143 (10)
C4B	0.0425 (12)	0.0276 (11)	0.0162 (11)	0.0013 (9)	−0.0049 (9)	0.0069 (9)
C5B	0.0391 (12)	0.0242 (10)	0.0192 (11)	0.0114 (9)	0.0033 (8)	0.0042 (9)
C6B	0.0221 (9)	0.0221 (9)	0.0175 (10)	0.0055 (7)	0.0021 (7)	0.0085 (8)
C7B	0.0210 (9)	0.0268 (10)	0.0253 (11)	0.0097 (8)	0.0058 (7)	0.0090 (9)
C8B	0.0245 (9)	0.0227 (10)	0.0260 (11)	0.0081 (8)	0.0018 (8)	0.0111 (9)
C9B	0.0151 (8)	0.0238 (9)	0.0197 (10)	0.0019 (7)	−0.0003 (7)	0.0106 (8)
C10B	0.0262 (10)	0.0271 (10)	0.0261 (12)	0.0016 (8)	−0.0015 (8)	0.0134 (9)
C11B	0.0343 (11)	0.0377 (12)	0.0190 (11)	−0.0039 (9)	0.0005 (8)	0.0159 (10)
C12B	0.0284 (10)	0.0389 (12)	0.0173 (11)	−0.0007 (9)	0.0047 (8)	0.0039 (10)
C13B	0.0236 (9)	0.0234 (10)	0.0190 (10)	0.0037 (8)	0.0019 (7)	0.0044 (9)
C14B	0.0171 (8)	0.0211 (9)	0.0152 (9)	0.0002 (7)	−0.0019 (6)	0.0075 (8)
C15B	0.0225 (9)	0.0168 (9)	0.0207 (10)	0.0057 (7)	−0.0012 (7)	0.0043 (8)
C16B	0.0196 (8)	0.0222 (9)	0.0246 (11)	0.0050 (7)	−0.0016 (7)	0.0067 (9)
C17B	0.0196 (8)	0.0245 (10)	0.0165 (10)	0.0040 (7)	−0.0011 (7)	0.0070 (8)
C18B	0.0446 (13)	0.0212 (10)	0.0376 (14)	0.0008 (9)	−0.0074 (10)	0.0091 (10)
C1C	0.0183 (8)	0.0215 (9)	0.0122 (9)	0.0083 (7)	0.0047 (6)	0.0057 (8)
C2C	0.0259 (10)	0.0262 (10)	0.0180 (10)	0.0046 (8)	−0.0012 (7)	0.0018 (9)
C3C	0.0382 (12)	0.0413 (13)	0.0169 (11)	0.0169 (10)	−0.0027 (8)	0.0047 (10)
C4C	0.0444 (13)	0.0361 (12)	0.0190 (11)	0.0194 (10)	0.0058 (9)	0.0148 (10)
C5C	0.0280 (10)	0.0276 (10)	0.0254 (12)	0.0098 (8)	0.0069 (8)	0.0140 (9)
C6C	0.0180 (8)	0.0235 (9)	0.0200 (10)	0.0086 (7)	0.0046 (7)	0.0098 (8)
C7C	0.0205 (9)	0.0223 (9)	0.0249 (11)	0.0030 (7)	0.0026 (7)	0.0092 (9)
C8C	0.0180 (8)	0.0292 (10)	0.0215 (11)	0.0030 (8)	−0.0008 (7)	0.0086 (9)
C9C	0.0200 (8)	0.0192 (9)	0.0173 (10)	0.0053 (7)	0.0010 (7)	0.0074 (8)
C10C	0.0348 (11)	0.0251 (10)	0.0158 (10)	0.0015 (8)	−0.0018 (8)	0.0043 (9)
C11C	0.0428 (12)	0.0258 (10)	0.0154 (10)	0.0113 (9)	0.0052 (8)	0.0063 (9)
C12C	0.0308 (10)	0.0292 (11)	0.0242 (11)	0.0114 (9)	0.0115 (8)	0.0142 (10)
C13C	0.0203 (9)	0.0277 (10)	0.0248 (11)	0.0033 (8)	0.0024 (7)	0.0111 (9)
C14C	0.0189 (8)	0.0188 (9)	0.0146 (9)	0.0053 (7)	0.0007 (6)	0.0069 (8)
C15C	0.0220 (9)	0.0178 (9)	0.0181 (10)	−0.0015 (7)	0.0032 (7)	0.0054 (8)
C16C	0.0194 (9)	0.0244 (10)	0.0258 (11)	0.0026 (7)	0.0045 (7)	0.0103 (9)
C17C	0.0200 (9)	0.0282 (10)	0.0180 (10)	0.0067 (8)	0.0030 (7)	0.0091 (9)
C18C	0.0495 (14)	0.0235 (11)	0.0339 (14)	0.0067 (10)	0.0088 (10)	0.0080 (11)
C1D	0.0152 (8)	0.0217 (9)	0.0135 (9)	0.0067 (7)	0.0010 (6)	0.0069 (8)
C2D	0.0221 (9)	0.0294 (10)	0.0208 (11)	0.0034 (8)	0.0017 (7)	0.0098 (9)
C3D	0.0273 (10)	0.0441 (13)	0.0239 (12)	0.0073 (9)	0.0065 (8)	0.0151 (11)
C4D	0.0355 (11)	0.0419 (13)	0.0136 (10)	0.0109 (10)	0.0048 (8)	0.0035 (10)
C5D	0.0286 (10)	0.0239 (10)	0.0167 (10)	0.0074 (8)	−0.0022 (7)	0.0009 (9)
C6D	0.0164 (8)	0.0199 (9)	0.0193 (10)	0.0083 (7)	0.0012 (7)	0.0043 (8)
C7D	0.0236 (9)	0.0218 (9)	0.0247 (11)	0.0013 (8)	−0.0017 (7)	0.0078 (9)
C8D	0.0214 (9)	0.0268 (10)	0.0257 (11)	0.0025 (8)	0.0035 (8)	0.0083 (9)
C9D	0.0223 (9)	0.0257 (10)	0.0197 (10)	0.0062 (8)	0.0047 (7)	0.0079 (9)
C10D	0.0392 (12)	0.0273 (11)	0.0263 (12)	0.0011 (9)	0.0059 (9)	0.0147 (10)
C11D	0.0516 (14)	0.0262 (11)	0.0211 (12)	0.0126 (10)	0.0021 (9)	0.0101 (10)
C12D	0.0329 (11)	0.0331 (11)	0.0190 (11)	0.0150 (9)	−0.0022 (8)	0.0050 (10)
C13D	0.0227 (9)	0.0242 (10)	0.0186 (10)	0.0055 (8)	0.0007 (7)	0.0048 (9)
C14D	0.0221 (9)	0.0209 (9)	0.0138 (9)	0.0107 (7)	0.0050 (7)	0.0075 (8)
C15D	0.0199 (8)	0.0192 (9)	0.0174 (10)	0.0031 (7)	−0.0021 (7)	0.0060 (8)
C16D	0.0199 (9)	0.0244 (10)	0.0224 (10)	0.0040 (7)	−0.0014 (7)	0.0070 (9)
C17D	0.0205 (8)	0.0255 (10)	0.0178 (10)	0.0085 (7)	0.0024 (7)	0.0110 (8)
C18D	0.0617 (16)	0.0247 (11)	0.0344 (14)	0.0152 (11)	0.0128 (12)	0.0063 (11)

### Geometric parameters (Å, °)

**Table:**

N1A—C1A	1.420 (2)	C12B—H12B	0.9500
N1A—C14A	1.425 (2)	C13B—C14B	1.406 (3)
N1A—C15A	1.471 (2)	C13B—H13B	0.9500
N2A—C18A	1.481 (3)	C15B—C16B	1.521 (2)
N2A—C17A	1.493 (3)	C15B—H15C	0.9900
N2A—H2AB	0.9200	C15B—H15D	0.9900
N2A—H2AC	0.9200	C16B—C17B	1.519 (3)
N1B—C1B	1.424 (2)	C16B—H16C	0.9900
N1B—C14B	1.429 (2)	C16B—H16D	0.9900
N1B—C15B	1.470 (2)	C17B—H17C	0.9900
N2B—C18B	1.483 (2)	C17B—H17D	0.9900
N2B—C17B	1.493 (2)	C18B—H18D	0.9800
N2B—H2BB	0.9200	C18B—H18E	0.9800
N2B—H2BC	0.9200	C18B—H18F	0.9800
N1C—C1C	1.421 (2)	C1C—C2C	1.406 (3)
N1C—C14C	1.428 (2)	C1C—C6C	1.411 (3)
N1C—C15C	1.471 (2)	C2C—C3C	1.381 (3)
N2C—C18C	1.478 (3)	C2C—H2CA	0.9500
N2C—C17C	1.497 (2)	C3C—C4C	1.387 (3)
N2C—H2CB	0.9200	C3C—H3CA	0.9500
N2C—H2CC	0.9200	C4C—C5C	1.382 (3)
N1D—C1D	1.422 (2)	C4C—H4CA	0.9500
N1D—C14D	1.428 (2)	C5C—C6C	1.396 (3)
N1D—C15D	1.467 (2)	C5C—H5CA	0.9500
N2D—C18D	1.482 (3)	C6C—C7C	1.532 (3)
N2D—C17D	1.489 (2)	C7C—C8C	1.524 (3)
N2D—H2DB	0.9200	C7C—H7CA	0.9900
N2D—H2DC	0.9200	C7C—H7CB	0.9900
C1A—C2A	1.409 (3)	C8C—C9C	1.501 (3)
C1A—C6A	1.411 (3)	C8C—H8CA	0.9900
C2A—C3A	1.369 (3)	C8C—H8CB	0.9900
C2A—H2AA	0.9500	C9C—C10C	1.390 (3)
C3A—C4A	1.386 (3)	C9C—C14C	1.396 (2)
C3A—H3AA	0.9500	C10C—C11C	1.394 (3)
C4A—C5A	1.393 (3)	C10C—H10C	0.9500
C4A—H4AA	0.9500	C11C—C12C	1.386 (3)
C5A—C6A	1.393 (3)	C11C—H11C	0.9500
C5A—H5AA	0.9500	C12C—C13C	1.386 (3)
C6A—C7A	1.525 (3)	C12C—H12C	0.9500
C7A—C8A	1.524 (3)	C13C—C14C	1.403 (3)
C7A—H7AA	0.9900	C13C—H13C	0.9500
C7A—H7AB	0.9900	C15C—C16C	1.525 (3)
C8A—C9A	1.508 (3)	C15C—H15E	0.9900
C8A—H8AA	0.9900	C15C—H15F	0.9900
C8A—H8AB	0.9900	C16C—C17C	1.519 (3)
C9A—C10A	1.391 (3)	C16C—H16E	0.9900
C9A—C14A	1.396 (3)	C16C—H16F	0.9900
C10A—C11A	1.393 (3)	C17C—H17E	0.9900
C10A—H10A	0.9500	C17C—H17F	0.9900
C11A—C12A	1.388 (3)	C18C—H18G	0.9800
C11A—H11A	0.9500	C18C—H18H	0.9800
C12A—C13A	1.387 (3)	C18C—H18I	0.9800
C12A—H12A	0.9500	C1D—C6D	1.406 (3)
C13A—C14A	1.399 (3)	C1D—C2D	1.408 (3)
C13A—H13A	0.9500	C2D—C3D	1.377 (3)
C15A—C16A	1.523 (3)	C2D—H2DA	0.9500
C15A—H15A	0.9900	C3D—C4D	1.394 (3)
C15A—H15B	0.9900	C3D—H3DA	0.9500
C16A—C17A	1.525 (3)	C4D—C5D	1.383 (3)
C16A—H16A	0.9900	C4D—H4DA	0.9500
C16A—H16B	0.9900	C5D—C6D	1.400 (3)
C17A—H17A	0.9900	C5D—H5DA	0.9500
C17A—H17B	0.9900	C6D—C7D	1.528 (3)
C18A—H18A	0.9800	C7D—C8D	1.528 (3)
C18A—H18B	0.9800	C7D—H7DA	0.9900
C18A—H18C	0.9800	C7D—H7DB	0.9900
C1B—C2B	1.395 (3)	C8D—C9D	1.500 (3)
C1B—C6B	1.403 (2)	C8D—H8DA	0.9900
C2B—C3B	1.390 (3)	C8D—H8DB	0.9900
C2B—H2BA	0.9500	C9D—C10D	1.389 (3)
C3B—C4B	1.379 (3)	C9D—C14D	1.393 (3)
C3B—H3BA	0.9500	C10D—C11D	1.394 (3)
C4B—C5B	1.390 (3)	C10D—H10D	0.9500
C4B—H4BA	0.9500	C11D—C12D	1.390 (3)
C5B—C6B	1.392 (3)	C11D—H11D	0.9500
C5B—H5BA	0.9500	C12D—C13D	1.390 (3)
C6B—C7B	1.506 (3)	C12D—H12D	0.9500
C7B—C8B	1.524 (3)	C13D—C14D	1.402 (2)
C7B—H7BA	0.9900	C13D—H13D	0.9500
C7B—H7BB	0.9900	C15D—C16D	1.531 (3)
C8B—C9B	1.524 (3)	C15D—H15G	0.9900
C8B—H8BA	0.9900	C15D—H15H	0.9900
C8B—H8BB	0.9900	C16D—C17D	1.520 (3)
C9B—C10B	1.405 (3)	C16D—H16G	0.9900
C9B—C14B	1.407 (3)	C16D—H16H	0.9900
C10B—C11B	1.383 (3)	C17D—H17G	0.9900
C10B—H10B	0.9500	C17D—H17H	0.9900
C11B—C12B	1.377 (3)	C18D—H18J	0.9800
C11B—H11B	0.9500	C18D—H18K	0.9800
C12B—C13B	1.386 (3)	C18D—H18L	0.9800
			
C1A—N1A—C14A	117.77 (14)	C17B—C16B—C15B	113.78 (15)
C1A—N1A—C15A	118.55 (15)	C17B—C16B—H16C	108.8
C14A—N1A—C15A	117.12 (15)	C15B—C16B—H16C	108.8
C18A—N2A—C17A	112.49 (16)	C17B—C16B—H16D	108.8
C18A—N2A—H2AB	109.1	C15B—C16B—H16D	108.8
C17A—N2A—H2AB	109.1	H16C—C16B—H16D	107.7
C18A—N2A—H2AC	109.1	N2B—C17B—C16B	112.18 (16)
C17A—N2A—H2AC	109.1	N2B—C17B—H17C	109.2
H2AB—N2A—H2AC	107.8	C16B—C17B—H17C	109.2
C1B—N1B—C14B	117.82 (14)	N2B—C17B—H17D	109.2
C1B—N1B—C15B	115.96 (15)	C16B—C17B—H17D	109.2
C14B—N1B—C15B	119.30 (15)	H17C—C17B—H17D	107.9
C18B—N2B—C17B	112.37 (16)	N2B—C18B—H18D	109.5
C18B—N2B—H2BB	109.1	N2B—C18B—H18E	109.5
C17B—N2B—H2BB	109.1	H18D—C18B—H18E	109.5
C18B—N2B—H2BC	109.1	N2B—C18B—H18F	109.5
C17B—N2B—H2BC	109.1	H18D—C18B—H18F	109.5
H2BB—N2B—H2BC	107.9	H18E—C18B—H18F	109.5
C1C—N1C—C14C	118.46 (14)	C2C—C1C—C6C	118.56 (17)
C1C—N1C—C15C	119.45 (15)	C2C—C1C—N1C	119.15 (16)
C14C—N1C—C15C	116.07 (15)	C6C—C1C—N1C	122.28 (16)
C18C—N2C—C17C	113.03 (16)	C3C—C2C—C1C	121.42 (19)
C18C—N2C—H2CB	109.0	C3C—C2C—H2CA	119.3
C17C—N2C—H2CB	109.0	C1C—C2C—H2CA	119.3
C18C—N2C—H2CC	109.0	C2C—C3C—C4C	120.6 (2)
C17C—N2C—H2CC	109.0	C2C—C3C—H3CA	119.7
H2CB—N2C—H2CC	107.8	C4C—C3C—H3CA	119.7
C1D—N1D—C14D	118.17 (14)	C5C—C4C—C3C	118.02 (19)
C1D—N1D—C15D	118.84 (15)	C5C—C4C—H4CA	121.0
C14D—N1D—C15D	116.60 (15)	C3C—C4C—H4CA	121.0
C18D—N2D—C17D	113.21 (17)	C4C—C5C—C6C	123.36 (19)
C18D—N2D—H2DB	108.9	C4C—C5C—H5CA	118.3
C17D—N2D—H2DB	108.9	C6C—C5C—H5CA	118.3
C18D—N2D—H2DC	108.9	C5C—C6C—C1C	117.98 (18)
C17D—N2D—H2DC	108.9	C5C—C6C—C7C	116.04 (17)
H2DB—N2D—H2DC	107.7	C1C—C6C—C7C	125.95 (17)
C2A—C1A—C6A	118.40 (18)	C8C—C7C—C6C	117.86 (16)
C2A—C1A—N1A	119.65 (17)	C8C—C7C—H7CA	107.8
C6A—C1A—N1A	121.95 (16)	C6C—C7C—H7CA	107.8
C3A—C2A—C1A	122.1 (2)	C8C—C7C—H7CB	107.8
C3A—C2A—H2AA	119.0	C6C—C7C—H7CB	107.8
C1A—C2A—H2AA	119.0	H7CA—C7C—H7CB	107.2
C2A—C3A—C4A	120.3 (2)	C9C—C8C—C7C	111.71 (15)
C2A—C3A—H3AA	119.9	C9C—C8C—H8CA	109.3
C4A—C3A—H3AA	119.9	C7C—C8C—H8CA	109.3
C3A—C4A—C5A	118.1 (2)	C9C—C8C—H8CB	109.3
C3A—C4A—H4AA	120.9	C7C—C8C—H8CB	109.3
C5A—C4A—H4AA	120.9	H8CA—C8C—H8CB	107.9
C4A—C5A—C6A	123.1 (2)	C10C—C9C—C14C	118.87 (17)
C4A—C5A—H5AA	118.4	C10C—C9C—C8C	122.76 (17)
C6A—C5A—H5AA	118.4	C14C—C9C—C8C	118.36 (17)
C5A—C6A—C1A	117.93 (17)	C9C—C10C—C11C	121.05 (19)
C5A—C6A—C7A	116.16 (17)	C9C—C10C—H10C	119.5
C1A—C6A—C7A	125.90 (18)	C11C—C10C—H10C	119.5
C8A—C7A—C6A	118.24 (16)	C12C—C11C—C10C	119.80 (19)
C8A—C7A—H7AA	107.8	C12C—C11C—H11C	120.1
C6A—C7A—H7AA	107.8	C10C—C11C—H11C	120.1
C8A—C7A—H7AB	107.8	C13C—C12C—C11C	119.95 (18)
C6A—C7A—H7AB	107.8	C13C—C12C—H12C	120.0
H7AA—C7A—H7AB	107.1	C11C—C12C—H12C	120.0
C9A—C8A—C7A	111.64 (16)	C12C—C13C—C14C	120.19 (18)
C9A—C8A—H8AA	109.3	C12C—C13C—H13C	119.9
C7A—C8A—H8AA	109.3	C14C—C13C—H13C	119.9
C9A—C8A—H8AB	109.3	C9C—C14C—C13C	120.12 (17)
C7A—C8A—H8AB	109.3	C9C—C14C—N1C	118.27 (16)
H8AA—C8A—H8AB	108.0	C13C—C14C—N1C	121.59 (16)
C10A—C9A—C14A	119.11 (17)	N1C—C15C—C16C	110.18 (15)
C10A—C9A—C8A	123.12 (17)	N1C—C15C—H15E	109.6
C14A—C9A—C8A	117.78 (17)	C16C—C15C—H15E	109.6
C9A—C10A—C11A	121.01 (19)	N1C—C15C—H15F	109.6
C9A—C10A—H10A	119.5	C16C—C15C—H15F	109.6
C11A—C10A—H10A	119.5	H15E—C15C—H15F	108.1
C12A—C11A—C10A	119.6 (2)	C17C—C16C—C15C	113.63 (15)
C12A—C11A—H11A	120.2	C17C—C16C—H16E	108.8
C10A—C11A—H11A	120.2	C15C—C16C—H16E	108.8
C13A—C12A—C11A	120.12 (18)	C17C—C16C—H16F	108.8
C13A—C12A—H12A	119.9	C15C—C16C—H16F	108.8
C11A—C12A—H12A	119.9	H16E—C16C—H16F	107.7
C12A—C13A—C14A	120.18 (18)	N2C—C17C—C16C	112.27 (16)
C12A—C13A—H13A	119.9	N2C—C17C—H17E	109.2
C14A—C13A—H13A	119.9	C16C—C17C—H17E	109.2
C9A—C14A—C13A	119.99 (18)	N2C—C17C—H17F	109.2
C9A—C14A—N1A	118.42 (16)	C16C—C17C—H17F	109.2
C13A—C14A—N1A	121.56 (16)	H17E—C17C—H17F	107.9
N1A—C15A—C16A	110.20 (14)	N2C—C18C—H18G	109.5
N1A—C15A—H15A	109.6	N2C—C18C—H18H	109.5
C16A—C15A—H15A	109.6	H18G—C18C—H18H	109.5
N1A—C15A—H15B	109.6	N2C—C18C—H18I	109.5
C16A—C15A—H15B	109.6	H18G—C18C—H18I	109.5
H15A—C15A—H15B	108.1	H18H—C18C—H18I	109.5
C15A—C16A—C17A	114.88 (15)	C6D—C1D—C2D	118.84 (17)
C15A—C16A—H16A	108.5	C6D—C1D—N1D	122.21 (16)
C17A—C16A—H16A	108.5	C2D—C1D—N1D	118.95 (17)
C15A—C16A—H16B	108.5	C3D—C2D—C1D	121.6 (2)
C17A—C16A—H16B	108.5	C3D—C2D—H2DA	119.2
H16A—C16A—H16B	107.5	C1D—C2D—H2DA	119.2
N2A—C17A—C16A	111.45 (16)	C2D—C3D—C4D	120.0 (2)
N2A—C17A—H17A	109.3	C2D—C3D—H3DA	120.0
C16A—C17A—H17A	109.3	C4D—C3D—H3DA	120.0
N2A—C17A—H17B	109.3	C5D—C4D—C3D	118.64 (19)
C16A—C17A—H17B	109.3	C5D—C4D—H4DA	120.7
H17A—C17A—H17B	108.0	C3D—C4D—H4DA	120.7
N2A—C18A—H18A	109.5	C4D—C5D—C6D	122.69 (19)
N2A—C18A—H18B	109.5	C4D—C5D—H5DA	118.7
H18A—C18A—H18B	109.5	C6D—C5D—H5DA	118.7
N2A—C18A—H18C	109.5	C5D—C6D—C1D	118.16 (18)
H18A—C18A—H18C	109.5	C5D—C6D—C7D	115.69 (17)
H18B—C18A—H18C	109.5	C1D—C6D—C7D	126.14 (17)
C2B—C1B—C6B	119.63 (18)	C6D—C7D—C8D	118.03 (17)
C2B—C1B—N1B	122.30 (17)	C6D—C7D—H7DA	107.8
C6B—C1B—N1B	118.05 (15)	C8D—C7D—H7DA	107.8
C3B—C2B—C1B	120.78 (19)	C6D—C7D—H7DB	107.8
C3B—C2B—H2BA	119.6	C8D—C7D—H7DB	107.8
C1B—C2B—H2BA	119.6	H7DA—C7D—H7DB	107.1
C4B—C3B—C2B	119.80 (18)	C9D—C8D—C7D	111.84 (15)
C4B—C3B—H3BA	120.1	C9D—C8D—H8DA	109.2
C2B—C3B—H3BA	120.1	C7D—C8D—H8DA	109.2
C3B—C4B—C5B	119.8 (2)	C9D—C8D—H8DB	109.2
C3B—C4B—H4BA	120.1	C7D—C8D—H8DB	109.2
C5B—C4B—H4BA	120.1	H8DA—C8D—H8DB	107.9
C4B—C5B—C6B	121.41 (19)	C10D—C9D—C14D	118.83 (18)
C4B—C5B—H5BA	119.3	C10D—C9D—C8D	122.82 (18)
C6B—C5B—H5BA	119.3	C14D—C9D—C8D	118.36 (17)
C5B—C6B—C1B	118.61 (17)	C9D—C10D—C11D	121.26 (19)
C5B—C6B—C7B	123.12 (18)	C9D—C10D—H10D	119.4
C1B—C6B—C7B	118.27 (17)	C11D—C10D—H10D	119.4
C6B—C7B—C8B	111.72 (16)	C12D—C11D—C10D	119.54 (19)
C6B—C7B—H7BA	109.3	C12D—C11D—H11D	120.2
C8B—C7B—H7BA	109.3	C10D—C11D—H11D	120.2
C6B—C7B—H7BB	109.3	C11D—C12D—C13D	120.05 (19)
C8B—C7B—H7BB	109.3	C11D—C12D—H12D	120.0
H7BA—C7B—H7BB	107.9	C13D—C12D—H12D	120.0
C9B—C8B—C7B	117.28 (16)	C12D—C13D—C14D	119.87 (18)
C9B—C8B—H8BA	108.0	C12D—C13D—H13D	120.1
C7B—C8B—H8BA	108.0	C14D—C13D—H13D	120.1
C9B—C8B—H8BB	108.0	C9D—C14D—C13D	120.44 (17)
C7B—C8B—H8BB	108.0	C9D—C14D—N1D	118.45 (16)
H8BA—C8B—H8BB	107.2	C13D—C14D—N1D	121.10 (16)
C10B—C9B—C14B	117.99 (18)	N1D—C15D—C16D	110.32 (15)
C10B—C9B—C8B	115.28 (17)	N1D—C15D—H15G	109.6
C14B—C9B—C8B	126.71 (17)	C16D—C15D—H15G	109.6
C11B—C10B—C9B	122.8 (2)	N1D—C15D—H15H	109.6
C11B—C10B—H10B	118.6	C16D—C15D—H15H	109.6
C9B—C10B—H10B	118.6	H15G—C15D—H15H	108.1
C12B—C11B—C10B	118.6 (2)	C17D—C16D—C15D	114.46 (15)
C12B—C11B—H11B	120.7	C17D—C16D—H16G	108.6
C10B—C11B—H11B	120.7	C15D—C16D—H16G	108.6
C11B—C12B—C13B	120.49 (19)	C17D—C16D—H16H	108.6
C11B—C12B—H12B	119.8	C15D—C16D—H16H	108.6
C13B—C12B—H12B	119.8	H16G—C16D—H16H	107.6
C12B—C13B—C14B	121.35 (19)	N2D—C17D—C16D	111.78 (16)
C12B—C13B—H13B	119.3	N2D—C17D—H17G	109.3
C14B—C13B—H13B	119.3	C16D—C17D—H17G	109.3
C13B—C14B—C9B	118.67 (18)	N2D—C17D—H17H	109.3
C13B—C14B—N1B	119.09 (17)	C16D—C17D—H17H	109.3
C9B—C14B—N1B	122.24 (16)	H17G—C17D—H17H	107.9
N1B—C15B—C16B	110.75 (14)	N2D—C18D—H18J	109.5
N1B—C15B—H15C	109.5	N2D—C18D—H18K	109.5
C16B—C15B—H15C	109.5	H18J—C18D—H18K	109.5
N1B—C15B—H15D	109.5	N2D—C18D—H18L	109.5
C16B—C15B—H15D	109.5	H18J—C18D—H18L	109.5
H15C—C15B—H15D	108.1	H18K—C18D—H18L	109.5
			
C14A—N1A—C1A—C2A	−125.96 (18)	C14C—N1C—C1C—C2C	129.01 (18)
C15A—N1A—C1A—C2A	24.9 (2)	C15C—N1C—C1C—C2C	−22.6 (2)
C14A—N1A—C1A—C6A	53.9 (2)	C14C—N1C—C1C—C6C	−52.1 (2)
C15A—N1A—C1A—C6A	−155.25 (16)	C15C—N1C—C1C—C6C	156.23 (16)
C6A—C1A—C2A—C3A	−2.3 (3)	C6C—C1C—C2C—C3C	3.1 (3)
N1A—C1A—C2A—C3A	177.58 (18)	N1C—C1C—C2C—C3C	−178.01 (17)
C1A—C2A—C3A—C4A	1.7 (3)	C1C—C2C—C3C—C4C	−0.8 (3)
C2A—C3A—C4A—C5A	−0.3 (3)	C2C—C3C—C4C—C5C	−1.7 (3)
C3A—C4A—C5A—C6A	−0.3 (3)	C3C—C4C—C5C—C6C	2.1 (3)
C4A—C5A—C6A—C1A	−0.4 (3)	C4C—C5C—C6C—C1C	0.1 (3)
C4A—C5A—C6A—C7A	178.84 (19)	C4C—C5C—C6C—C7C	−178.10 (18)
C2A—C1A—C6A—C5A	1.6 (3)	C2C—C1C—C6C—C5C	−2.7 (2)
N1A—C1A—C6A—C5A	−178.26 (17)	N1C—C1C—C6C—C5C	178.46 (16)
C2A—C1A—C6A—C7A	−177.52 (18)	C2C—C1C—C6C—C7C	175.35 (17)
N1A—C1A—C6A—C7A	2.6 (3)	N1C—C1C—C6C—C7C	−3.5 (3)
C5A—C6A—C7A—C8A	−175.18 (17)	C5C—C6C—C7C—C8C	173.64 (17)
C1A—C6A—C7A—C8A	4.0 (3)	C1C—C6C—C7C—C8C	−4.4 (3)
C6A—C7A—C8A—C9A	−60.4 (2)	C6C—C7C—C8C—C9C	61.0 (2)
C7A—C8A—C9A—C10A	−109.5 (2)	C7C—C8C—C9C—C10C	111.0 (2)
C7A—C8A—C9A—C14A	70.0 (2)	C7C—C8C—C9C—C14C	−70.1 (2)
C14A—C9A—C10A—C11A	1.0 (3)	C14C—C9C—C10C—C11C	0.3 (3)
C8A—C9A—C10A—C11A	−179.4 (2)	C8C—C9C—C10C—C11C	179.19 (18)
C9A—C10A—C11A—C12A	−1.4 (3)	C9C—C10C—C11C—C12C	0.9 (3)
C10A—C11A—C12A—C13A	1.3 (3)	C10C—C11C—C12C—C13C	−1.0 (3)
C11A—C12A—C13A—C14A	−0.7 (3)	C11C—C12C—C13C—C14C	0.0 (3)
C10A—C9A—C14A—C13A	−0.5 (3)	C10C—C9C—C14C—C13C	−1.3 (3)
C8A—C9A—C14A—C13A	179.97 (18)	C8C—C9C—C14C—C13C	179.71 (17)
C10A—C9A—C14A—N1A	−178.46 (17)	C10C—C9C—C14C—N1C	177.03 (16)
C8A—C9A—C14A—N1A	2.0 (3)	C8C—C9C—C14C—N1C	−1.9 (2)
C12A—C13A—C14A—C9A	0.3 (3)	C12C—C13C—C14C—C9C	1.2 (3)
C12A—C13A—C14A—N1A	178.24 (17)	C12C—C13C—C14C—N1C	−177.09 (17)
C1A—N1A—C14A—C9A	−73.3 (2)	C1C—N1C—C14C—C9C	72.2 (2)
C15A—N1A—C14A—C9A	135.44 (17)	C15C—N1C—C14C—C9C	−135.26 (17)
C1A—N1A—C14A—C13A	108.7 (2)	C1C—N1C—C14C—C13C	−109.53 (19)
C15A—N1A—C14A—C13A	−42.5 (2)	C15C—N1C—C14C—C13C	43.1 (2)
C1A—N1A—C15A—C16A	66.8 (2)	C1C—N1C—C15C—C16C	−70.4 (2)
C14A—N1A—C15A—C16A	−142.22 (16)	C14C—N1C—C15C—C16C	137.35 (16)
N1A—C15A—C16A—C17A	−177.75 (16)	N1C—C15C—C16C—C17C	177.12 (16)
C18A—N2A—C17A—C16A	−165.59 (16)	C18C—N2C—C17C—C16C	173.74 (16)
C15A—C16A—C17A—N2A	−65.2 (2)	C15C—C16C—C17C—N2C	63.4 (2)
C14B—N1B—C1B—C2B	−109.8 (2)	C14D—N1D—C1D—C6D	52.6 (2)
C15B—N1B—C1B—C2B	41.0 (2)	C15D—N1D—C1D—C6D	−156.35 (16)
C14B—N1B—C1B—C6B	71.6 (2)	C14D—N1D—C1D—C2D	−127.45 (18)
C15B—N1B—C1B—C6B	−137.62 (17)	C15D—N1D—C1D—C2D	23.6 (2)
C6B—C1B—C2B—C3B	0.2 (3)	C6D—C1D—C2D—C3D	−2.4 (3)
N1B—C1B—C2B—C3B	−178.37 (17)	N1D—C1D—C2D—C3D	177.64 (17)
C1B—C2B—C3B—C4B	0.9 (3)	C1D—C2D—C3D—C4D	0.3 (3)
C2B—C3B—C4B—C5B	−1.8 (3)	C2D—C3D—C4D—C5D	1.7 (3)
C3B—C4B—C5B—C6B	1.6 (3)	C3D—C4D—C5D—C6D	−1.7 (3)
C4B—C5B—C6B—C1B	−0.5 (3)	C4D—C5D—C6D—C1D	−0.5 (3)
C4B—C5B—C6B—C7B	178.7 (2)	C4D—C5D—C6D—C7D	178.58 (18)
C2B—C1B—C6B—C5B	−0.4 (3)	C2D—C1D—C6D—C5D	2.5 (2)
N1B—C1B—C6B—C5B	178.22 (17)	N1D—C1D—C6D—C5D	−177.62 (15)
C2B—C1B—C6B—C7B	−179.63 (18)	C2D—C1D—C6D—C7D	−176.46 (17)
N1B—C1B—C6B—C7B	−1.0 (3)	N1D—C1D—C6D—C7D	3.4 (3)
C5B—C6B—C7B—C8B	109.6 (2)	C5D—C6D—C7D—C8D	−175.50 (16)
C1B—C6B—C7B—C8B	−71.2 (2)	C1D—C6D—C7D—C8D	3.5 (3)
C6B—C7B—C8B—C9B	59.9 (2)	C6D—C7D—C8D—C9D	−59.9 (2)
C7B—C8B—C9B—C10B	176.24 (17)	C7D—C8D—C9D—C10D	−109.9 (2)
C7B—C8B—C9B—C14B	−2.1 (3)	C7D—C8D—C9D—C14D	69.8 (2)
C14B—C9B—C10B—C11B	1.8 (3)	C14D—C9D—C10D—C11D	1.2 (3)
C8B—C9B—C10B—C11B	−176.79 (19)	C8D—C9D—C10D—C11D	−179.01 (19)
C9B—C10B—C11B—C12B	0.4 (3)	C9D—C10D—C11D—C12D	−1.7 (3)
C10B—C11B—C12B—C13B	−0.6 (3)	C10D—C11D—C12D—C13D	1.0 (3)
C11B—C12B—C13B—C14B	−1.3 (3)	C11D—C12D—C13D—C14D	0.0 (3)
C12B—C13B—C14B—C9B	3.5 (3)	C10D—C9D—C14D—C13D	−0.2 (3)
C12B—C13B—C14B—N1B	−176.75 (17)	C8D—C9D—C14D—C13D	−179.94 (17)
C10B—C9B—C14B—C13B	−3.6 (3)	C10D—C9D—C14D—N1D	−178.64 (17)
C8B—C9B—C14B—C13B	174.75 (18)	C8D—C9D—C14D—N1D	1.6 (3)
C10B—C9B—C14B—N1B	176.65 (17)	C12D—C13D—C14D—C9D	−0.5 (3)
C8B—C9B—C14B—N1B	−5.0 (3)	C12D—C13D—C14D—N1D	177.96 (17)
C1B—N1B—C14B—C13B	128.10 (18)	C1D—N1D—C14D—C9D	−72.2 (2)
C15B—N1B—C14B—C13B	−21.6 (2)	C15D—N1D—C14D—C9D	136.13 (18)
C1B—N1B—C14B—C9B	−52.2 (2)	C1D—N1D—C14D—C13D	109.35 (19)
C15B—N1B—C14B—C9B	158.11 (16)	C15D—N1D—C14D—C13D	−42.3 (2)
C1B—N1B—C15B—C16B	140.25 (16)	C1D—N1D—C15D—C16D	67.2 (2)
C14B—N1B—C15B—C16B	−69.5 (2)	C14D—N1D—C15D—C16D	−141.31 (16)
N1B—C15B—C16B—C17B	178.11 (16)	N1D—C15D—C16D—C17D	−176.53 (16)
C18B—N2B—C17B—C16B	173.14 (16)	C18D—N2D—C17D—C16D	−166.99 (16)
C15B—C16B—C17B—N2B	63.2 (2)	C15D—C16D—C17D—N2D	−64.0 (2)

### Hydrogen-bond geometry (Å, °)

**Table:**

*D*—H···*A*	*D*—H	H···*A*	*D*···*A*	*D*—H···*A*
N2A—H2AB···Cl3^i^	0.92	2.22	3.1186 (16)	165
N2A—H2AC···Cl3	0.92	2.17	3.0811 (17)	169
N2B—H2BB···Cl1	0.92	2.20	3.1027 (16)	168
N2B—H2BC···Cl4	0.92	2.20	3.1014 (16)	165
N2C—H2CB···Cl1	0.92	2.20	3.1069 (16)	167
N2C—H2CC···Cl4	0.92	2.21	3.1065 (16)	166
N2D—H2DB···Cl2	0.92	2.22	3.1176 (16)	166
N2D—H2DC···Cl2^ii^	0.92	2.18	3.0859 (16)	168

Symmetry codes: (i) −*x*+1, −*y*+1, −*z*+1; (ii) −*x*+1, −*y*+1, −*z*+2.
